# Effect of cyclodextrin glucosyltransferase extracted from *Bacillus xiaoxiensis* on wheat dough and bread properties

**DOI:** 10.3389/fnut.2022.1026678

**Published:** 2022-11-01

**Authors:** Lianzhan Yang, Jinxin Cai, Haifeng Qian, Yan Li, Hui Zhang, Xiguang Qi, Li Wang, Guoliang Cao

**Affiliations:** ^1^State Key Laboratory of Food Science and Technology, School of Food Science and Technology, Jiangnan University, Wuxi, China; ^2^Lingquegu Biotechnology Co., Ltd., Quanzhou, China; ^3^Jiangsu Daddy Sweety Food Technology Co., Ltd., Wuxi, China

**Keywords:** cyclodextrin glucosyltransferase, wheat dough, dough rheology, bread properties, bread staling

## Abstract

In this study, the cyclodextrin glucosyltransferase (CGTase) was extracted from *Bacillus xiaoxiensis*. CGTase had negative effects on dough viscoelastic properties and gluten strength but had positive effects on bread baking qualities and anti-staling properties. Adding an appropriate amount of CGTase (less than 0.3 U/g) could improve the specific volume, crumb texture, crust color, moisture content, and crumb hardness of bread. The bread crumb with 0.4 U/g CGTase (based on flour weight) had the lowest retrogradation enthalpy of 0.53 ± 0.10 J/g and the lowest relative crystallinity of 16.1%, which indicated the alleviating effect of amylopectin crystallization. Moreover, CGTase reduced the moisture from forming crystal lattices and limited starch molecule migration. The T_2_ transverse relaxation results showed that the increase of immobilized water content in the bread with CGTase was lower than the control after 5 days of storage, which implied the water-holding capacity of the bread was enhanced and provided information on the inhibition of water migration. Hence, the CGTase could be a potential bread improver.

## Introduction

Bread is an important staple food in people’s daily life. The staling process of bread is a common problem in the baking industry and has to be solved. During the storage period, bread quality will deteriorate due to starch retrogradation, such as crumb firming, reduction of moisture content, and loss of fresh bread flavor. In the world, there are huge economic losses and wasting food because of bread staling. Usually, it’s a good way to add improvers such as enzymes to enhance the baking quality of bread and delay the staling rate. The enzyme preparations (α-amylase, lipase, xylanase, etc.) can improve bread’s specific volume and moisture content while decreasing crumb hardness (1, 2). The role of α-amylase is to catalyze the hydrolysis of α-1, 4-glycosidic bonds in starch molecules (amylose and amylopectin), producing maltodextrins and oligosaccharides. Lipase in baking catalyzes the decomposition of fat in the dough to produce some small molecules, such as acylglycerols, free fatty acids, and so on, which help to form a more stable gluten network. Xylanase is a hydrolase that can attack the arabinoxylan backbone and destroy glycosidic bonds, resulting in changing the function and physicochemical properties of arabinoxylan.

In this study, we chose CGTase which was extracted from *Bacillus xiaoxiensis* to investigate its impact on the wheat dough and bread properties. CGTase belongs to the α-amylase family and is a multifunctional enzyme that can catalyze four enzymatic reactions: hydrolysis, cyclization, coupling, and disproportionation (3–5). In the food industry, CGTase is mainly manifested as an enzyme that can use starch to produce cyclodextrin (CD) through a cyclization reaction. According to the number of glucose units, CD mainly includes α-, β-, and γ-cyclodextrin (containing 6, 7, and 8 glucose units, respectively). *Bacillus xiaoxiensis* is a Gram-stain-positive, slightly halophilic, catalase-positive, oxidase-negative, endospore-forming, motile, facultatively anaerobic, rod-shaped bacterium, which was found in China Xiaoxi National Nature Reserve (6). Its genome sequence contains the gene cluster of CGTase, indicating that it has the potential to produce CGTase (7).

The specific volume of baked products significantly increased when the CGTase was added because the swelling capacity and solubility of wheat starch granules increased (8). Furthermore, CGTase can not only reduce the hardness of the fresh bread crumb but also slow down the firming process of the crumb during storage (4). Several works have shown that the bread staling retarded by CGTase could be attributed to the CGTase effectively hydrolyzing starch and producing CD or other low molecule weight carbohydrates, leading to the inhibition of amylopectin crystallization. Meanwhile, the CD may form complexes with fats/oils in the flour to hinder the retrogradation of amylopectin (9, 10).

CD enhanced the water absorption capacity of flour because CD had a large number of hydroxyl groups that could form hydrogen bonds with water molecules, thereby limiting the availability of water and affecting the formation of the gluten network (11). Because of this, it could cause changes in the mixing properties and extensogram properties of the dough. Furthermore, CD could increase the ratio of α-helix in gluten protein and decrease the ratio of β-sheet, indicating that the secondary structure of gluten protein has changed under the action of CD (12). However, the influence of CD on the gluten network is related to its concentration. A low level of CD slightly improved the quality of the gluten network, while a high level of CD decomposed the gluten network and caused a decrease in the specific volume of bread (13).

This study aimed to explore the potential applications of the CGTase in the food industry. There have been many reports about CGTase used in bread, but it is not comprehensive and detailed. Hence, in order to further understand the important role of CGTase on wheat dough and bread, this study investigates the effect of CGTase extracted from *Bacillus xiaoxiensis* on the rheological properties of dough, the baking quality, and the aging process of bread crumbs.

## Materials and methods

### Materials

The ingredients for the bread doughs were: Golden mountain bread wheat flour (protein 12.8% and ash 0.52%, Yihai Kerry Ltd., China), saf-Instant (Lesaffre, Company, China), cyclodextrin glucosyltransferase (*Bacillus xiaoxiensis* CGTase, EC 2.4.1.19, 50 U/mL, extracted by School of Food Science and Technology, Jiangnan University).

The inoculum was standardized to obtain a suspension with lyophilized cells (107 UFC/mL) and was grown in 250 mL of liquid culture medium at pH 10. The culture was incubated at 37°C with a continuous stirring at 150 rpm for 48 h. Subsequently, the culture was divided into 50 mL portions, transferred to a new flask containing 1,000 mL of liquid medium, and kept at 37°C with a continuous stirring at 80 rpm for 5 days. The cells were removed by centrifugation at 8,800 × *g* for 10 min. A saturated ammonium sulfate (80%) was added to the cell-free supernatant to precipitate the protein. The mixture was kept at 4°C for 48 h, and the resulting precipitate was separated by centrifugation at 4°C for 20 min. Afterward, it was dissolved in 50 mol/L Tris-HCl buffer. It was purified by biospecific affinity chromatography using Sepharose 6B gel and β-CD as a binder and concentrated by ultrafiltration in small volume CENTRICON micro-concentrators (Amicon) (Millipore Corporation, Billerica, MA) to obtain purified and concentrated CGTase.

### Preparation of dough and bread samples

Dough samples were prepared without yeast addition based on the following formula: 1,000 g of flour, 12.0 g of salt, 100.0 g of sucrose, 60.0 g of shortening (Nanqiao Food Ltd., China), and 600.0 g of water. Bread samples were prepared by straight dough procedure using the above baking formula and 10.0 g of compressed yeast. Firstly, the solids were mixed evenly, then water and shortening were added and optimally mixed. After the dough was rested for 45 min, the dough was divided into several pieces of 150 g and proofed for 50 min at 36°C and 80% RH. Baking was achieved in an electric oven at 180°C of the upper heater and 200°C of the bottom heater for 25 min. The recipe was baked with no addition of CGTase (control bread) and with the addition of 0.1, 0.2, 0.3, and 0.4 U/g of CGTase (based on flour weight). The CGTase was added by mixing with the wheat flour. In addition, the dough and bread samples were placed into PA + PE plastic films and stored at 20 ± 2°C and 60 ± 2% RH for 7 days.

### Dough properties

#### Rheological properties of dough

Rheological properties of dough without yeast addition were determined by AR G2 rheometer (TA Instrument, Company, American) (14). The experiment was equipped with a 4-cm parallel plate fixture and tested at 25°C. An appropriate amount of samples was taken from the center of the dough and placed under the jig; then, the test gap was adjusted to 2 mm. Furthermore, the excess sample was trimmed off with a plastic spatula, and the exposed edges of the dough were covered with silicone oil to avoid evaporation of water. A preliminary strain sweep test at 1 Hz showed that the storage moduli (*G’*) and loss moduli (*G”*) were constant until 0.02% (linear viscoelastic domain). Under a constant shear strain of 0.02% (in the linear viscoelastic region), an oscillation experiment with a frequency sweep range of 0.1–10 Hz was performed. The dynamic rheological properties of the samples were evaluated by the *G’*, *G”* and loss angle tanδ (*G”*/*G’*).

#### Farinogram properties and extensogram properties

The farinogram properties and extensogram properties of the wheat dough were estimated by using Brabender Farinograph and Brabender Extensograph (Duisburg, Germany) according to the method of AACC 54–10 (15). After the farinograph was idling to zero, 300 g flour and 30–120 U CGTase were added to the kneading bowl. Then, after the stirring rotor started, water was immediately injected into the kneading bowl. The farinograph was used to obtain water absorption, dough development time, dough stability, and degree of softening. The addition level of water must be controlled until the peak midline of the silty curve within the range of 500 ± 20 FU. Also, the water must be added within 25 s.

In the extensograph test, the farinograph equipped with S 300 H mixer was used to prepare dough with constant consistency (500 FU) (16). The mixing time of the dough was constant at 5 min. Moreover, the dough was given a cylindrical shape and placed in a proving cabinet for 45, 90, and 135 min. The extensograph was used to record energy, maximum resistance (R), extensibility (E), and ratio figure (R/E).

### Bread properties

#### Loaf specific volume

According to the method of AACC 10-05.01 (15), the specific volume of bread was determined by replacing the millet seeds. The determination was carried out by placing a known volume of millet seeds in a suitable container. The specific volume was calculated by dividing the volume (mL) by the weight (g) of bread (mL/g or cm^3^/g).

#### Moisture content

The moisture content of the bread crumb was determined by oven drying at 105°C until constant weight according to the method of AACC 44-15A (15). Moisture was determined on a crumb cylinder (40 mm diameter) taken from the center of each slice after 0, 1, 3, 5, and 7 days of storage.

#### Crumb texture analysis

After 0, 1, 3, 5, and 7 days of storage, bread was analyzed for crumb firmness as described by the previously developed procedure (1) with some modification. A slice of bread with a height of 20 mm was used, and a compression test was performed by using a TA-XT2i texture analyzer (Stable Micro System™ Co., Godalming, UK) equipped with the 25 mm diameter cylindrical probe (P/25). The bread samples were compressed at a pretest speed of 1 mm/s, test speed of 1 mm/s, and post speed of 2 mm/s with a trigger load of 5 g for compressing the center of the sliced bread to 40% of its original height. The recorded peak force was reported as crumb firmness. For each storage duration, 6 bread were analyzed in total.

#### Color measurement

The color of fresh bread crust (the center of the outmost layer) and crumb (the internal center) was measured by Chromameter (Konica Minolta CR-400, Japan) equipped with a built-in C illuminant to obtain the value of L*(the brightness, 0 = black, 100 = white), a*(the red-greenness, + value = red, –value = green), b*(the yellow-blueness, + value = yellow, –value = blue), and ΔE (color difference). The corrected chroma meter was adjusted to the CIE-L*a*b* color space.

#### Crumb structure

The internal texture structure of the sliced bread was evaluated by using an image scanner (Canon iR-ADV 4225, Japan), and Image J software (NIH, Bethesda, USA) was used for photos analysis (17). The resulting image was first resized to a 3 × 3 cm^2^ image from the center of sliced bread, then converted into an 8-bit grayscale binary. Finally, the threshold-divided was used to calculate the cells density (numbers/9 cm^2^), the average area of cells (mm^2^), and crumb porosity (cells area/9 cm^2^).

### Bread retrogradation measurement

#### Differential scanning calorimetry analysis

Bread thermal properties were measured by a SIINT instrument (X-DSC 700, Japan), and the retrogradation enthalpy (ΔH, J/g) was analyzed by Universal Analysis 3.9A software (TA Instruments, DE, USA) to indicate the degree of starch retrogradation (5, 10). After 7 days of storage at 20°C, the central bread crumb was freeze-dried, milled into powder, and passed through a 120-mesh sieve. Afterward, about 3 mg of the crumb powders was accurately weighed in an aluminum pan, 6 μL of deionized water was added, and hermetically sealed. All the pans were equilibrated overnight at 4°C, and an empty pan was used as a reference. The samples were heated from 20 to 90°C at a rate of 10°C/min. Five measurements of each bread-making replication were performed.

#### X-ray diffraction analysis

The Bruker D8-Advance X-ray diffraction (XRD) instrument (Bruker AXS Inc., Germany) equipped with nickel-filtered Cu-Kα (wavelength 1.5405 Å) was used to determine the starch crystallinity during the storage of bread. The samples were treated with the same method as mentioned in section “Differential scanning calorimetry analysis.” The radiation working condition was 40 kV and 30 mA; the diffractograms were recorded from 5° to 45° at a scan speed of 2°/min. The crystallinity (%) was processed and calculated by MDI Jade 6.0 software (Materials Data, Inc., Livermore, CA, USA), according to Palacios et al. (2).

#### Low field nuclear magnetic resonance

Proton relaxation measurements were measured using a MesoMR23-060V–I NMR spectrometer (Suzhou Niumai Electronic Technology Co., Ltd., China) equipped with a 25-mm-diameter NMR probe at 25 ± 0.1°C. Approximately 1.5 g of the crumb was taken from the center of the bread after storage for 2 h and 5 days, placed into a 10 mm NMR tube, and sealed with polytetrafluoroethylene sealing tape to prevent water loss. ^1^H transverse relaxation time (^1^H T_2_) experiment was carried out with a Carr-Purcell-Meiboom-Gill (CPMG) pulse sequence. The experimental parameters were set as follows: the duration between successive scans (TW) was 500 ms, and the echo time (TE) was 0.25 ms. The data from 2,000 echoes (NECH) were acquired through 8 scan repetitions (NS). The measurement results were calculated using the MultiExp Inv Analysis software (Niumag, China).

### Statistical analysis

Each experiment was repeated at least three times. The results were expressed as the means ± standard deviations (SD) and analyzed by one-way analysis of variance (ANOVA). The means were compared by Duncan’s multiple range test using the SPSS 25.0 statistical software program (SPSS Inc., Chicago, USA). The differences were considered significant at *P* < 0.05.

## Results and discussion

### Dough properties

#### Rheological properties

**FIGURE 1:**
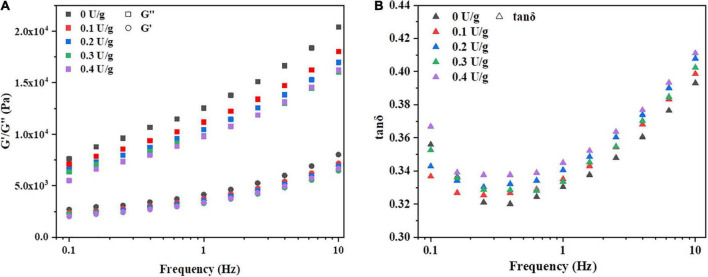
Variation in **(A)** storage modulus (G’), loss modulus (G”), and **(B)** tan δ of dough with or without CGTase.

The effect of CGTase on storage modulus (*G’*), loss modulus (*G”*), and tanδ of dough with a frequency sweep from 0.1 to 10 Hz is shown in Figure 1. The *G’* moduli’s value is higher than the value of *G”* moduli, and the tanδ value is less than 1, indicating that the dough could exhibit a solid-like behavior. A similar phenomenon was reported by Onyango et al. (14), who stated that batter supplemented with α-amylase had solid viscoelastic properties when the storage modulus was predominant over the loss modulus. This was possibly due to CGTase could hydrolyze the α-(14) glycosidic bonds of starch to produce low molecular weight oligosaccharides, resulting in the lower resistance of the dough (18). As shown in Figure 1A, both *G’* and *G”* increased with an increase in frequency for all samples, but tanδ decreased first and then increased in Figure 1B. All samples with CGTase had lower *G’* and *G”* moduli than the control dough. Also, the viscoelasticity of the dough decreased when CGTase was added. The dough with CGTase has a lower softness and poorer elongation properties, indicating that it might be unfavorable for dough handling and mechanical processing in the actual production process. When the addition amount of CGTase was at lower levels, amylase would hydrolyze the internal chain segments of starch, leading to the residual amylopectin, the produced small molecular starch clusters and malto-oligosaccharides may reduce the viscosity of the dough (19). However, the tanδ of doughs with CGTase were all higher than the control dough; it might imply that CGTase weakened the dough’s solid-like behavior, making it more liquid-like. The results suggested that water absorption and distribution of the dough were changed after the starch is hydrolyzed into CD and other low MW oligosaccharides. The bound water level was reduced, possibly due to CD having a lower water-binding capacity than damaged starch (20).

#### Farinogram properties and extensogram properties

Characterizing the rheological properties of dough can effectively predict processing behavior and control food quality. It can be seen from Table 1 that when CGTase was added, the values of dough development time and stability time decreased, while the degree of softening increased, indicating that CGTase accelerated the dough development process and destroyed the capacity of dough to resist stirring. The degree of softening reflected the capacity of the dough to be subjected to mechanical shearing force. Therefore, a higher degree of softening means a poorer gluten network structure (21). It could be due to CGTase hydrolyzed starch and destructed the gluten structure, resulting in a reduced consistency of dough. This made the dough difficult to shape during processing and easily collapsed in the final product (22). Meanwhile, the disintegrated structure of gluten might be the reason for the decrease in flour water absorption. The weakened gluten network might reduce the interactions between water and amine groups of proteins, as well as the hydroxyl and carbonyl groups of the starch. Amylase would increase the damaged starch content in the dough, which was positively correlated with flour water absorption. However, there were no significant differences in dough development time and stability time among all groups with different CGTase levels (*P* > 0.05), indicating that the effect of CGTase on gluten was fairly limited. Thus, the results suggested that the dough containing CGTase had poor water retaining capacity and rheological properties, which confirmed the previous research related to amylase.

**TABLE 1:** Farinograph and extensograph analysis of doughs without or with CGTase.

CGTase levels (U/g)	Flour water absorption (%)	Dough development time (min)	Stability (min.)	Degree of softening (FU)	Proving 45 min			
					
					Energy (cm^2^)	Maximum resistance R_m_ (mm)	Extensibility (mm)	Ratio R_m_/E
Control	69.35 ± 0.07^b^	12.75 ± 0.78^a^	15.50 ± 0.57^b^	50.00 ± 2.83^a^	186.00 ± 4.24^a^	714.50 ± 16.26^a^	209.50 ± 14.44^a^	3.40 ± 0.28^a^
0.1	69.53 ± 0.21^b^	12.07 ± 0.12^a^	14.20 ± 0.87^a^	51.33 ± 5.13^a^	167.50 ± 16.26^a^	702.50 ± 10.61^a^	199.00 ± 7.07^a^	3.55 ± 0.07^a^
0.2	68.75 ± 0.21^a^	12.40 ± 0.57^a^	13.65 ± 0.34^a^	54.50 ± 1.71^a^	163.00 ± 4.24^a^	726.50 ± 43.13^a^	183.00 ± 1.41^a^	4.00 ± 0.28^a^
0.3	68.75 ± 0.49^a^	12.20 ± 0.57^a^	12.70 ± 0.14^a^	58.50 ± 2.12^ab^	182.50 ± 17.68^a^	818.00 ± 16.97^b^	190.50 ± 31.82^a^	4.30 ± 0.15^a^
0.4	68.63 ± 0.15^a^	12.37 ± 0.15^a^	13.10 ± 0.20^a^	61.67 ± 1.15^b^	−	−	−	−

**CGTase levels** **(U/g)**	**Proving 90 min.**				**Proving 135 min**			
		
	**Energy** **(cm^2^)**	**Maximum resistance** **R_m_ (mm)**	**Extensibility** **(mm)**	**Ratio** **R_m_/E**	**Energy** **(cm^2^)**	**Maximum resistance** **R_m_ (mm)**	**Extensibility** **(mm)**	**Ratio** **R_m_/E**

Control	178.50 ± 19.09^b^	922.50 ± 31.82^a^	184.50 ± 7.78^c^	5.05 ± 0.07^a^	173.50 ± 17.68^c^	968.50 ± 53.03^b^	178.50 ± 4.95^b^	5.40 ± 0.14^a^
0.1	163.50 ± 13.44^b^	909.00 ± 48.08^a^	156.00 ± 7.07^b^	5.80 ± 0.00^b^	119.00 ± 12.73^ab^	829.00 ± 4.24^a^	114.50 ± 10.61^a^	7.25 ± 0.64^b^
0.2	111.50 ± 7.78^a^	838.50 ± 21.92^a^	116.00 ± 7.07^a^	7.25 ± 0.21^c^	100.50 ± 6.36^a^	836.50 ± 19.09^a^	104.00 ± 8.49^a^	8.05 ± 0.49^b^
0.3	162.00 ± 8.49^b^	1082.00 ± 32.53^b^	131.00 ± 1.41^a^	8.25 ± 0.21^d^	135.50 ± 9.19^b^	1012.00 ± 29.70^b^	121.00 ± 5.66^a^	8.35 ± 0.64^b^

Means in the same column with different superscript lowercase letters indicate a significant difference (*P* < 0.05).

CGTase, cyclodextrin glucosyltransferase (based on flour weight); Control, dough in the absence of CGTase.

The extensogram results of dough with or without CGTase after proving 45, 90, and 135 min are shown in Table 1. When the amount of CGTase was constant, the energy of the dough gradually decreased with an increase in proofing time. Noticeably, the lowest energy loss was recorded in the control dough, indicating that the dough strength was damaged after the addition of CGTase. This was consistent with the results of the farinogram test. Likewise, CGTase significantly decreased the extensibility of the dough after proofing for 90 and 135 min, indicating that the transverse extensibility of the dough was weakened. In the same proofing time, the maximum resistance of the dough showed a decreasing trend first and then increased with the increase of the CGTase addition level. This finding suggests that the enzymes that can hydrolyze starch must be added in an appropriate amount. Bread with α-amylase could produce dextrin, but excessive dextrin would enhance the viscosity of bread crumb and affect the taste, so the amount of α-amylase should be added in low levels (23). Therefore, when the level of CGTase is low, the elasticity of the dough would reduce due to the weakened gluten structure, resulting in a deterioration of the anti-stretch characteristic of dough. While when the amount of CGTase is high, the starch in the dough would be effectively hydrolyzed to CD or other oligosaccharides, leading to a remarkable increase in the viscosity of the dough. It was correlated to the study that by adding high amounts of amylase, the values of tanδ and *G’* would decrease, but the viscosity of the dough would increase (24). So, the resistance to deformation tended to increase after that. Considering the two parameters of maximum resistance and extensibility, there was a significant difference (*P* < 0.05) in maximum resistance to extensibility (R_m_/E) between the doughs with and without CGTase, explaining that the CGTase slightly improved the extensogram properties of doughs.

### Bread properties

It can be observed from Table 2 that the specific volume of the bread with CGTase was larger than the control bread since yeast may use the low molecular weight carbohydrates that were hydrolyzed from starch to generate a large amount of carbon dioxide, increasing the number of cells and the area of cells in crumb (4, 10, 25). In addition, CD had a hydrophobic cavity which helps to form a complex with hydrophobin to improve the solubility of hydrophobin. Cyclodextrin-protein complex participates in the formation of the gluten network, which can better retain carbon dioxide (13, 26).

**TABLE 2:** Baking characteristics of bread without or with different addition amounts of CGTase.

CGTase levels (U/g)	Specific volume (cm^3^/g)	Bread crust color			Cell parameters			Crumb Δ H (J/g)
			
		L*	a*	b*	Cells density (number/cm^2^)	Mean area of cells (mm^2^)	Crumb porosity (%)	
Control	4.33 ± 0.12^a^	68.63 ± 0.65^d^	12.31 ± 1.41^a^	28.60 ± 1.95^a^	52.13 ± 4.55^a^	0.50 ± 0.08^a^	26.07 ± 3.02^a^	1.49 ± 0.04^d^
0.1	4.40 ± 0.14^ab^	67.85 ± 0.49^cd^	12.80 ± 1.12^a^	28.03 ± 1.76^a^	54.96 ± 4.04^a^	0.53 ± 0.10^a^	29.23 ± 1.29^b^	1.15 ± 0.11^c^
0.2	4.56 ± 0.15^b^	65.48 ± 1.04^b^	13.23 ± 1.71^a^	27.96 ± 1.98^a^	57.81 ± 7.20^a^	0.52 ± 0.17^a^	29.95 ± 2.40^b^	0.81 ± 0.14^b^
0.3	4.95 ± 0.14^c^	66.20 ± 1.72^bc^	13.90 ± 0.96^a^	31.26 ± 1.55^ab^	56.44 ± 6.24^a^	0.53 ± 0.04^a^	29.75 ± 2.38^b^	0.70 ± 0.03^ab^
0.4	4.59 ± 0.08^b^	63.04 ± 1.39^a^	16.17 ± 1.82^b^	32.76 ± 1.83^b^	62.33 ± 9.71^a^	0.49 ± 0.05^a^	30.78 ± 0.78^b^	0.53 ± 0.10^a^

Means in the same column with different superscript lowercase letters indicate a significant difference (*P* < 0.05).

CGTase, cyclodextrin glucosyltransferase (based on flour weight); Control, dough in the absence of CGTase.

As expected, the cells’ density and crumb porosity increased because of CGTase action, as shown in Table 2. The gas yield of yeast increased, which helped to improve the specific volume and softness of bread crumbs. In comparison, the specific volume of bread decreased when the level of CGTase reached 0.4 U/g flour. It might be due to the weakened gluten structure where some of the cell walls in the dough were broken, and carbon dioxide escaped into the environment, resulting in a decline in the gas-holding capacity of the dough. It has been reported that the increase of α-amylase activity had a remarkable effect on the specific volume of bread, but excessive α-amylase hydrolyzed a large amount of amylose and amylopectin during the baking process. So, it was difficult for the starch to form a gel during the cooling process, causing higher granule stickiness and poorer grain structure (27). There were the same characteristics as the decrease in the storage modulus and loss modulus of the dough during the rheological experiment.

The crust color was measured by determining the value of L*(brightness), a*(red-greenness), and b*(yellow-blueness). A significant decrease in L* value and an increase in a* value were noticed in the bread crust, indicating that the crust color got darker with the addition of CGTase. This change could be ascribed to CGTase produced reducing sugars such as glucose during the hydrolysis of starch, which provided the required energy for yeast and promoted the Maillard reaction in bread, resulting in a bread crust with a darker color.

**FIGURE 2:**
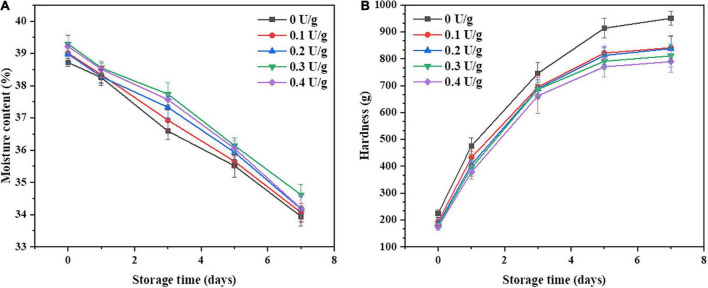
Moisture content **(A)** and hardness **(B)** of the bread with or without CGTase after storage at 20°C for 7 days.

Figures 2A,B shows the changes in crumb moisture content and crumb hardness of bread within 7 days of storage, respectively. There was an increase in the moisture content and a decrease in the hardness of the crumb when CGTase was added. Compared with the control, the bread with 0.4 U/g of CGTase exhibited the lowest hardness; also, the staling rate of the crumb decreased by 12.1% on the seventh day, proving that the CGTase has the potential to delay the hardening rate of crumb. It was reported that CGTase could reduce the hardness of bread crumbs during the storage period (4, 10, 25). The higher moisture content has contributed to maintaining the softness of the crumb. When the dough was developed in the farinogram test, the flour water absorption rate was decreased, while the moisture content of the bread was increased, as shown in Figure 2A. It may be due to the addition of CGTase, which reduced the moisture loss of bread during baking. Studies have shown that the water-binding abilities of proteins may be affected by the interaction between CDs and proteins through hydrogen bonding (11). In addition, the external parts of the CDs are hydrophilic and hence help water molecules and other components to form inclusion complexes, thereby improving the water absorption of the dough.

### Bread staling measurement

After the bread was stored for 7 days, the amylopectin would recrystallize due to retrogradation, which caused the value of enthalpy to change. In addition, the differential scanning calorimetry (DSC) was used to accurately measure the value of retrogradation enthalpy (ΔH) (5, 28). It can be seen from Table 2 that the control bread had the highest enthalpy value of 1.49 ± 0.04 J/g, while the bread with 0.4 U/g CGTase had the lowest enthalpy value of 0.53 ± 0.10 J/g. On the seventh day, the ΔH had a declining trend with the increase in CGTase levels. Therefore, it was speculated that the CGTase had the function of retarding bread staling. Moreover, CD could develop a complex with the lipids in the dough. The cyclodextrin-lipids complex has an emulsifying effect and also could interfere with the recrystallization of amylose (25, 29, 30). There were significant differences between the various groups (*P* < 0.05).

**FIGURE 3:**
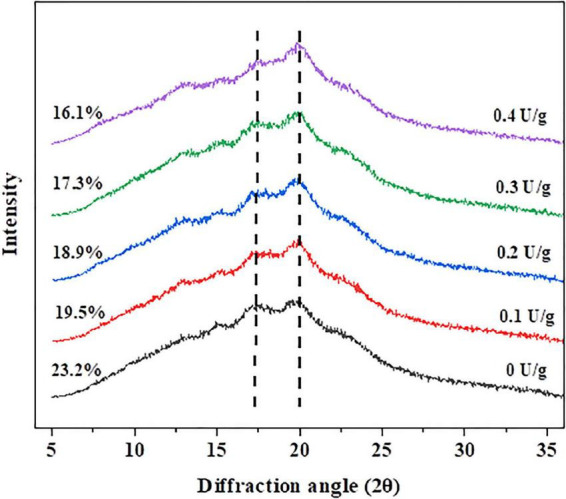
The X-ray diffraction patterns of wheat bread with different levels of CGTase after storage at 20°C for 7 days.

XRD was used to determine the crystallinity degree of the substance. So, it was effective to obtain the degree of retrogradation by measuring the crystallinity degree of amylose and amylopectin (31). After the bread was stored for 7 days, the recrystallization degree of the crumb is shown in Figure 3. There were two peaks at 17° and 20° diffraction angles (2θ), representing the B-type and V-type structures of starch crystals, respectively. During bread staling, the formation of the B-type crystalline pattern demonstrated the changes in the amorphous fraction and crystalline structures. The crystallinity of the control crumb was the highest (23.2%) on the seventh day of storage, while the bread crumb with 0.4 U/g CGTase was the lowest (16.1%). Likewise, with increasing of CGTase levels, the crystallinity of bread crumbs and the degree of starch retrogradation showed a downward tendency, indicating that CGTase had a retarding effect on the bread staling. The results could be attributed to two reasons. On the one hand, the decomposed starch was difficult to restore to the previous state; on the other hand, the intricate arrangement of dextrin produced by the enzyme might effectively interfere with the crystallization of starch (2, 23).

**FIGURE 4:**
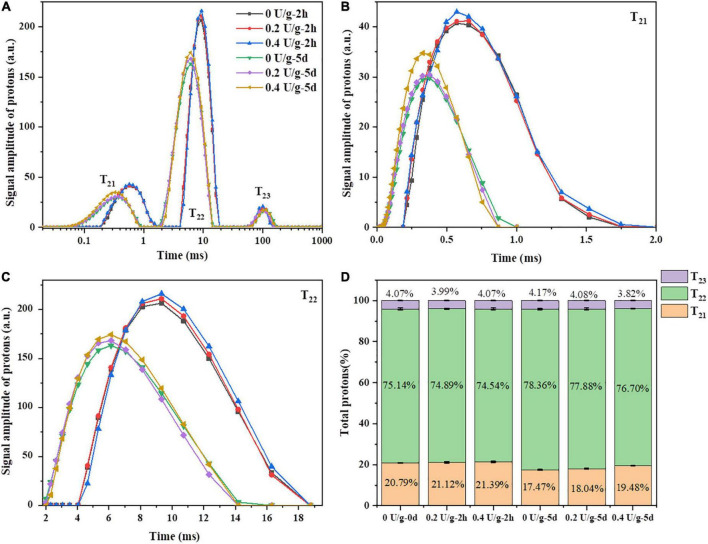
**(A)** The typical T_2_ relaxation time distribution curve of bread after 4 freeze–thaw cycles. **(B)** The partial enlarged drawing of T_21_. **(C)** The partial enlarged drawing of T_22_. **(D)** Peak area proportions of T_21_, T_22_ and T_23_.

Through measuring transverse relaxation times (T_2_), low field nuclear magnetic resonance (LF-NMR) was used to investigate the water state, distribution, and the degree of combination between water and various ingredients in bread during storage (32). As shown in Figure 4A, there were three peaks, i.e., T_21_ (0.01–2 ms), T_22_ (2–20 ms), and T_23_ (40–180 ms), corresponding to bound water, immobilized water, and free water, respectively. In Figures 4B and C, after 2 h of bread baking, there was a minor increase in the areas of T_21_ and T_22_ peaks of fresh bread when CGTase was added, indicating that the content of bound water and immobilized water in the bread was increased. That may be due to the depolymerization of starch by CGTase affecting the binding degree of water and starch macromolecules or gluten, thereby, the water migration may transform to low mobility and the baking loss would be reduced (28). The C_21_ of bread with CGTase increased significantly, indicating that the proportion of water combined with gluten protein increased, which might be the CDs produced by CGTase forming a complex with gluten protein, thereby forming more hydrogen bonds with water molecules and enhancing the water-holding capacity of gluten matrix (33). During the bread storage, there was a continuous downward trend of moisture content as indicated in Figure 2A. Meanwhile, the T_2_ of all bread showed a remarkable decline on the fifth day in Figure 4A. This was because the moisture would migrate from the bread crumb to the crust and finally evaporate into the external environment. On the other hand, the gelatinized starch would re-form an orderly crystalline structure when the starch was retrograded. The moisture combined with amorphous starch might be migrated to the crystallization area, so the crumb of the fifth day had a smaller ^1^H peak area and shorter T_2_ relaxation time.

It was reported that T_21_ represents the water combined with gluten, while T_22_ represents the water combined with starch or other polymers (34). On the fifth day, with the increase of CGTase levels, the T_22_ value of bread increased, indicating that water molecules bound to starch were less involved in the process of starch recrystallization and still had a longer relaxation time. Furthermore, as shown in Figure 4D, compared to the results of 0 day, all bread samples had a slight decrease in C_21_ and a slight increase in C_22_, indicating that the water might migrate from the gluten area to the starch area during storage. With the increase in CGTase levels, there was an increase in the number of protons of population T_21_ and a decrease in the number of protons of population T_22_ in the bread on the fifth day of storage. CGTase induced a water shift from free water to bound water or immobilized water, demonstrating that a large number of protons were involved in the formation of crystalline structures. Therefore, it could be inferred that the water in the gluten protein would continuously be fused into the starch crystal structure (35). In addition, the decreasing degree (3.32, 3.08, 1.91%) of the number of protons of population T_21_ and the increasing degree (3.22, 2.99, 2.16%) of the number of protons of population T_22_ decreased with the increase in CGTase levels, which could thus be suggested that the addition of CGTase would hinder moisture migration and retain more moisture staying in the gluten network. It might partly explain the reduced retrogradation rate of bread starch.

## Conclusion

This study provided insights into the influence of CGTase extracted from *Bacillus xiaoxiensis* on the wheat dough and bread quality. When the CGTase was added in high concentration, the doughs exhibited poor rheological properties and increased softening degree and viscosity, leading to difficulty in the shaping process. The CGTase in suitable levels (less than 0.3 U/g) could result in bread with a larger specific volume, higher moisture content, more cells, and softer crumbs. However, when the amount of CGTase was excessive, the starch was over-hydrolyzed, disrupting cell structure and decreasing air-holding capacity. Therefore, the specific volume of bread was reduced and exhibited a rough texture structure. Through DSC, XRD, and LF-NMR analysis, it was observed that the CGTase could reduce the retrogradation enthalpy, the relative crystallization of amylopectin, and the rate of water migration. This indicated that CGTase might delay bread staling and moisture loss. Because the CGTase hydrolyzed starch into small molecular polysaccharides, the damaged amylose and amylopectin could not regenerate into their original state. Moreover, the complex formed by the cyclodextrin and lipids and the cyclodextrin and protein might hinder moisture migration from the gluten area to the starch area, there by preventing starch retrogradation and water loss.

## Data availability statement

The original contributions presented in this study are included in the article/supplementary material, further inquiries can be directed to the corresponding author/s.

## Author contributions

LY: conceptualization, methodology, data curation, software, and writing—original draft preparation. JC: conceptualization and data curation. HQ: reviewing and editing. YL: data curation and project administration. HZ and XQ: validation. LW: conceptualization, reviewing and editing, funding acquisition, and writing—original draft preparation. GC: conceptualization, reviewing and editing, and funding acquisition. All authors contributed to the article and approved the submitted version.
